# Effects of atopy and rhinitis on exhaled nitric oxide values - a systematic review

**DOI:** 10.1186/2045-7022-1-8

**Published:** 2011-08-17

**Authors:** Daniela Linhares, Tiago Jacinto, Ana M Pereira, João A Fonseca

**Affiliations:** 1 Health Information and Decision Sciences, Faculty of Medicine of University of Porto, Alameda Hernâni Monteiro, 4200-319 Porto, Portugal; 2 CINTESIS, Faculty of Medicine of University of Porto, Alameda Hernâni Monteiro 4200-319 Porto, Portugal; 3 Allergy, Instituto & Hospital CUF Porto, 4460-188 Senhora da Hora, Portugal; 4 Immunoallergology, Centro Hospitalar S. João, Alameda Hernâni Monteiro, 4200 - 319 Porto Portugal

**Keywords:** Exhaled Nitric Oxide, Atopy, Rhinitis, Systematic Review

## Abstract

**Background:**

Atopy and rhinitis are among the factors affecting exhaled nitric oxide (FeNO) values and may contribute to difficulties in the clinical interpretation of FeNO measurements. However, data assessing their effects on FeNO values had never been summarized. This review aims to evaluate the effect of atopy and rhinitis in FeNO values in otherwise healthy individuals.

**Methods:**

A systematic review was performed in Pubmed, Scopus and ISI Web of Knowledge. A two-step selection process was completed, and from 2357 references 19 were included. The inclusion criteria were: participants without known diseases other than rhinitis; atopy assessement by SPT or Specific IgE; and FeNO measurements according to ATS/ERS recommendations.

**Results:**

The 8 articles measuring FeNO in children showed higher values in both allergic rhinitis and atopic children when compared with healthy children. The 11 articles performed in adults observed higher FeNO in AR patients comparatively with either healthy or atopic individuals. However, adult healthy and atopic individuals had similar FeNO values.

**Conclusions:**

FeNO values are higher in individuals with rhinitis and/or atopy without other health problems. These effects are small, seem to be independent and should be further studied using multivariate models. The effect of atopy was observed only in children. The combined effect of atopy and rhinitis produced higher FeNO values in adults. These results support that both atopy and rhinitis should be considered when interpreting or when defining FeNO reference values.

## Background

Exhaled nitric oxide (FeNO) is associated with asthma [1], and with airway eosinophilia [2]. Recently, it was suggested that FeNO production in the airways is under the influence of Th2 cytokines, IL-4 and IL-13, responsible for induction of Inducible Nitric Oxide Synthase (iNOS) expression in the airway epithelium, and thus for the increase of FeNO in inflammatory disorders [3].

Many individual factors influence FeNO values. In fact, the considerable inter-subject variability hampers the clinical interpretation of FeNO measurements [4,5]. Two important FeNO modifiers are atopy and rhinitis [6]. Some studies reported higher FeNO values in patients with atopy or allergic rhinitis alone [7-9], while others observed a relationship between elevated FeNO values and the exacerbation of rhinitis and the number of positive wheals on skin prick tests (SPT) [10,11]. However, results are controversial, as there are studies that did not observe significant differences of FeNO values in atopy or rhinitis [12-14].

The aim of this systematic review was to evaluate the effect of atopy and rhinitis in FeNO values in otherwise healthy individuals.

## Methods

This systematic review follows the PRISMA recommendations [15]. Three databases were searched: Pubmed, Scopus and ISI Web of Knowledge. The limits used were a) English language, b) publication date from 1990 to October 2009, and c) studies with original data. The main search terms were "FeNO", "Rhinitis, "Atopy" and equivalent expressions.

**Figure 1:**
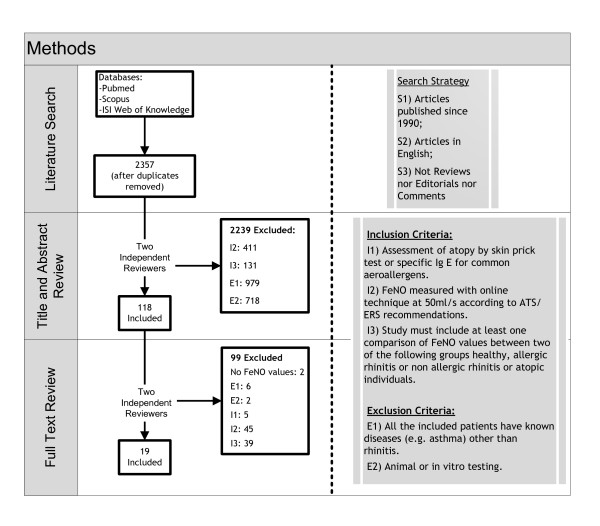
**Articles selection process**.

The inclusion criteria were: 1) atopy assessed by Skin Prick Tests (SPT) or specific IgE for common aeroallergens; 2) online FeNO measurements, according to the American Thoracic Society/European Respiratory Society (ATS/ERS) recommendations [6]; 3) study participants of at least two of the following groups: healthy, atopic, with allergic rhinitis or non allergic rhinitis. Exclusion criteria were 1) *in vitro *or animal experiments or 2) participants with known diseases other than rhinitis, e.g. asthma alone. A study group was defined as atopic in the presence of allergic sensitization without a medical diagnosis of asthma and rhinitis. Study selection had two phases (Figure 1). In Phase 1, two reviewers screened the titles and abstracts independently. If one of the reviewers included the abstract, it was allowed into the Phase 2. In Phase 2, manuscripts were analyzed independently and disagreements were discussed between reviewers

Data was extracted using an electronic form developed by the authors. Information on characteristics of the included studies and their samples was summarized in tables 1 and 2 and Additional File 1 (tables S1-S2). Six studies did not present FeNO values. Authors were contacted for the FeNO data, but no answer was obtained. Four of these studies had FeNO values comparisons and were included, and the remaining 2 were excluded.

**Table 1:** Description of the sample and methods of the studies included and how Atopy and Rhinitis were assessed

Article	Setting	**Sample Size**^**‡**^	Age	Sex (% of men)	Classification of:	
					
					*Atopic*	*Rhinits*
**Aronsson, 2005**	NI	43^†^	H: 41 (19-56); AR: 33 (18-55)^§^	11%	SPT	Symptoms

**Berlyne, 2000**	Outpatient clinics	131^†^	H: 36 (13); A: 37 (12)	43%	SPT	NA

**Cardinale, 2005**	Outpatient clinics (healthy: schools)	175^†^	H: 10.3 (2.2); AR: 10.1 (1.8)	29%	Specific IgE/Rast; Total IgE	Clinical Assessment

**Cibella, 2008**	NI	335	12.1 (10-16)^§^	43%	SPT	Symptoms

**Gratziou, 2008**	Outpatient clinics (healthy: volunteers)	27^†^	H: 36 (8); AR: 35 (10)	37%	SPT	Symptoms

**Hervás, 2008**	Outpatient clinics	90	10.8 (6-15)^§^	64%	SPT	Clinical Assessment

**Hung, 2007**	Outpatient clinics	60	5-14^||^	47%	Specific IgE	Symptoms

**Kosticas, 2008**	Schools	219*	H: 21.4 (2.3); AR: 21.8 (3.0)	52%	SPT	Symptoms

**Malmberg, 2006**	Schools	276*	11.6 (6.9-15.7)^§^	51%	SPT	NA

**Manson, 2009**	NI	20	H: 23-48; AR: 20-49^||^	20%	SPT	Symptoms

**Marcucci, 2007**	NI	41	6 -10^||^	NI	SPT; Specific IgE/Rast	NA

**Olin, 2004**	Workers	246^†^	H: 44.5 (0.69); AR: 41.5 (1.51)	94%	Specific IgE/Rast	Symptoms

**Prieto, 2002 (1)**	Outpatient clinics (Healthy: schools and volunteers)	48^†^	H: 28.6 (9.2); AR: 33.0 (10.5)	54%	SPT	Clinical Assessment

**Prieto, 2002 (2)**	Outpatient clinics (Healthy: schools and volunteers)	24^†^	H: 27.5 (3.3); AR: 34.9 (2.8)	33%	SPT	Clinical Assessment

**Profita, 2006**	NI	91	H: 10 (2.4); AR: 9.9 (2.9)	59%	SPT; Specific IgE/Rast; Total IgE	Clinical Assessment

**Rolla, 2007**	Outpatient clinics	108^†^	39.3 (11-75)^§^	45%	SPT	Symptoms

**Rouhos, 2008**	General Population	248^†^	45 (26-61)	72%	SPT	NI

**Saito, 2004**	NI	278	10-12^||^	50%	Specific IgE/Rast	NA

**Tanou, 2009**	NI	70*	H: 31.8 (5.9); AR: 32.8 (7.2)	46%	SPT; Total IgE	Symptoms

NI - No Information; NA: Not Applicable; A: Atopic; AR: Allergic Rhinitis; H: Healthy

* Sample includes smokers; ^† ^Sample of non-smoker individuals; ^‡^See more information in Additional File 1. Age values are present as Mean (SD), otherwise indicated. ^§^Mean (Range); ^||^Range.

**Table 2:** Comparison of FeNO values between groups in the 18 studies included

	Studies in Children (n = 8)								Studies in Adults (n = 11)										
	
	Mean (n = 4)			Median (n = 2)		Values not shown (n = 2)		Mean (n = 3)		Median (n = 6)						Values not shown (n = 2)			
	
	**Hervás 2008 **[8]	**Hung 2007 **[22]	**Malmberg 2006 **[21]	**Saito 2004 **[28]	**Cardinale 2005 **[11]	**Cibella 2008 **[12]	**Marcucci 2007 **[23]	**Profita 2006 **[13]	**Prieto 2002 **[29]	**Prieto, 2002 **[16]	**Rolla 2007 **[30]	**Rouhos 2008 **[31]	**Kostikas 2008 **[32]	**Tanou 2009 **[33]	**Berlyne 2000 **[14]	Gratziou **2008 **[34]	**Olin 2004 **[35]	**Aronsson 2005 **[36]	**Mansson 2009 **[37]
**FeNO Value**s																			

**Atopic**	19 (1.21)	-	14.6‡	33.3 (1.13)	-	17.4 (5.5-95.2)*	FeNO values not present^§^		-	-	-	13.2^†^	-	-	11 (6)	.	16.5 (11.3-27.6)	FeNO values not present^§^	
					
**AR**	26.3 (1.04)	24.68 (19.2)	-	-	15.3 (9.4-31.0)	19.1 (5.8-92.5)*			25.1 (5.7-102.9)^†^	DS: 63.1(1.26) OS: 30.2(1.27)	24.5 (3.32)	23.0 (11.6-43.6)*	17.0 (12.5-23.0)	20.5 (12.5-33)	-	12.5 (4.0-50.0)	31.0 (16.0-50.5)		
					
**NAR**	-	-	-	-	-	12.6 (2.8-71.8)*			-	-	16.1‡	-	-	-	-	-	19.2 (14.1-22.0)		
					
**Healthy**	7.9 (0.96)	9.44 (3.97)	10.3‡	15.1 (1.14)	5.9 (3.4-9.3)	13.1 (3.0-71.0)*			11.2 (5.0-31.5) **	12.6 (1.4)	13.5 (0.9)	15.5 †	10.5 (7.0-13.0)	9.5 (8.0-12.0)	9 (7)	3.8 (1.0-7.3)	15.8 (11.9-21.4)		

**Comparison of FeNO values between**^**||**^																			

**Atopic and Healthy**	↑↑	-	↑	↑↑	-	↑	-	-	-	-	-	NS (↓)	-	-	NS (↑)	-	NS (↑)	-	-

**AR and Healthy**	↑↑↑	↑↑↑	-	-	↑↑↑	NS (↑)	↑	NS (↔)	↑↑	E:↑↑↑ NE:↑↑	↑↑	NR (↑)	↑↑	↑↑	-	↑↑↑	NR (↑↑)	NS (↔)	↑

**NAR and Healthy**	-	-	-	-	-	NS (↓)	-	-	-	-	NS (↑)	-	-	-	-	-	NR (↑↑)	-	-

**AR and Atopic**	↑	-	-	-	-	NS (↑)	-	-	-	-	-	NR (↑↑)	-	-	-	-	↑↑	-	-

**AR and NAR**	-	-	-	-	-	↑	-	-	-	-	↑	-	-	-	-	-	↑	-	-

**Atopic and NAR**	-	-	-	-	-	NS (↑)	-	-	-	-	-	-	-	-	-	-	NR (↓)	-	-

Values are presented as Mean (SD) or as Median (Interquartil Range), otherwise indicated: *Median (Range), †Mean (Range), ‡ Dispersion Measure not presented, ^§^The studies' reports do not included numerical results. ^||^Comparison was based in a ratio between FeNO values in each group: 1, 2 or 3 arrows means a ratio increase/decrease on FeNO values of 1,2 or 3 or more times, respectively. DS: During Season OS: Out of Season. Adult classification was defined as a mean/median age value ≥18 years. Atopic was defined as presence of allergic sensitization without asthma and without rhinitis. All comparisons are statistically significant (p < 0.05), otherwise indicated. NR: Statistical significance not reported. NS: Not significant.

Two groups of studies were defined, based on the participants' age - less than 18 years old and adults. In each group, the studies were divided according to the summary measure used for reporting FeNO values. Four groups of FeNO values were defined: Allergic Rhinitis (AR), Non-Allergic Rhinitis (NAR), Healthy and Atopic. Values were compared in sets of 2, with a total of 6 comparisons. A ratio of FeNO values in each comparison is presented. One study provided results during and off pollen season [16]. Both results were included, in separate conditions. When data allowed it, meta-analysis were done using Review Manager 5 (Cochrane Collaboration) [17] using a random effects model, due to differences on studies methods. Results showed high heterogeneity (I^2^) and were not included for analysis (Additional File 2, figures S1-S3).

## Results

Articles search retrieved a total of 2357 references after duplicates' removal. In the first selection phase 2239 articles were excluded, mainly studies with patients with known conditions other than rhinitis (Figure 1). In the second selection phase, all full-text articles were retrieved and analyzed. Nineteen studies were included in the systematic review (Figure 1), in eight the participants were children and in 11 were adults. Most were cross sectional studies (n = 16), and 3 were prospective. Seven did not report the study setting, 8 recruited the subjects from outpatient clinics, 2 from schools, one from general population and another one from mills (Table 1). Sample sizes ranged from 20 to 276 participants (Table 1). Three studies included smokers and 9 included only non-smokers individuals; the remaining 7 studies did not report smoking status (Additional File 1, Table S1); one of the studies with children [11] excluded those that were exposed to tobacco smoke in the family.

Most studies used SPT to identify atopic patients (Table 1). Rhinitis was defined based on symptoms (n = 9) or clinical assessment (n = 5) (Table 1). The definition of healthy individuals was not reported in three studies, and all the others excluded atopic individuals (Additional File 1, Table S2).

**Figure 2:**
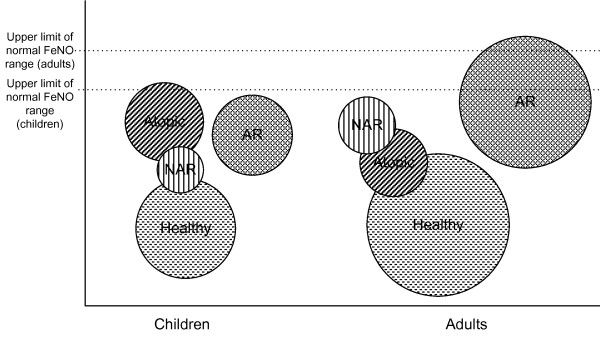
**Visualization of summaries of FeNO comparisons**. Groups with overlapping circles showed similar FeNO values. The radius of each circle is proportional to the number of articles measuring FeNO in each group. Upper limit of normal FeNO range was based on previous reports on children [21] and adults' [20] FeNO values. Atopic was defined as presence of allergic sensitization without asthma and without rhinitis.

Comparisons between defined groups were reported in all studies, but in 4 these comparisons did not present numerical values (Table 2). Seven articles compared Atopic and Healthy individuals. The 3 studies performed in adults showed no significant differences in FeNO values, while in children, the studies showed higher FeNO values in the atopic groups. Sixteen articles compared FeNO values between Allergic Rhinitis and Healthy individuals in a total of 17 comparisons; 12 showed significant higher values of FeNO in the AR patients. Two studies compared Atopic and Non-Allergic Rhinitis individuals: one performed in children observed a non-significant higher in FeNO values in atopic individuals, while another, with an adult population, observed lower values. When comparing Allergic Rhinitis and Atopic individuals all the 4 studies (2 in children) showed higher FENO values in allergic rhinitis; in 2 of them the values were significantly higher and in the other the statistical significance was not reported. All articles comparing Allergic Rhinitis and Non-Allergic Rhinitis subjects (n = 3) showed higher FeNO values in allergic rhinitis patients. From the three articles comparing FeNO values between Non-Allergic Rhinitis individuals and Healthy ones, 2 had no significant differences, and one showed higher values in the NAR group (Figure 2).

The collected data was insufficient for quantitative synthesis. For each comparison, 2 to 3 studies could be used, and 2 out of the 3 meta-analysis had high heterogeneity (I^2 ^= 100%) (Additional File 2, Figures S1-S3).

## Discussion

This is the first systematic review that summarizes the influence of Allergic Rhinitis and Atopy on FeNO values. FeNO was higher in children with atopy and in children with allergic rhinitis, when compared with children without rhinitis, atopic or with NAR. In adults, a similar increase was observed with AR, but FeNO values were similar between atopic and healthy individuals. These results are in agreement with previous non-systematic reviews on AR effect on nasal and/or exhaled nitric oxide values [5,18]. Previous results also support the consistent effect of atopy on FeNO values that we observed in healthy children; however they are not in agreement with the absence of effect of atopy in FeNO levels that we found in adults [5,19]. Similar values were also observed between atopic individuals without rhinitis and patients with NAR and with NAR and healthy children. However, only 3 studies with patients with NAR were available.

This systematic review supports the argument that atopy and rhinitis should be considered for the clinical interpretation of FeNO measurements. In fact, adults with allergic rhinitis and children with atopy (those presenting higher FeNO levels) may have FeNO measurements out of the 'normal' range previously proposed that do not take in account both atopy and rhinitis. To allow a visual representation of comparisons between groups we present the illustration in Figure 2[20,21].

Our study has some limitations. The interpretation of quantitative synthesis of data was not possible. FeNO values were presented in different ways (e.g. different summary and dispersion measures) (Table 2) so only 2 to 3 articles could be used for each comparison. Moreover, a high heterogeneity was observed (Additional File 2, Figure S1-S3). Despite our best efforts, we had no access to the values of FeNO in four studies. These articles were included on the review, allowing a broad qualitative assessment of published studies but could not be used in the meta-analysis.

The methods of the included studies were highly variable. There were variations in the definitions of atopy, rhinitis and healthy individuals. The definition of healthy individuals was particularly variable, often incomplete and sometimes missing. Also, various settings, eligibility criteria and sample sizes were used. In some of the studies with children [8,12,21-23] the age range was too broad (> 5 years). FeNO in children is strongly associated with age and body size [5]. The inclusion criteria regarding smoking status were different and many did not report the smoking status (Additional File 1, Table S1). As smoking [9,24-26], and passive smoking [27], can interfere with FeNO values, not accounting for the smoking status may be one of the reasons for the differences observed between the studies. Moreover, most studies presented only univariate analysis, with few multivariate models reported. Multivariate models could consider additional personal characteristics (e.g. height, age or gender).

Future studies should report adequate summary and dispersion measures, allowing for further quantitative synthesis. Samples sizes and subjects characteristics should be accurately described. Moreover, the factors that are known to modify FeNO should be taken into account. It is also important to follow current recommendations on FENO measurement [6].

In conclusion, FeNO values are higher in individuals with rhinitis and/or atopy without other diseases. These effects are small, seem to be independent and should be further studied using multivariate models. The causes for the effects of atopy and rhinitis on FENO values also need further study. The effect of atopy was observed only in children. The combined effect of atopy and rhinitis produced higher FeNO values in adults. These results support that atopy and rhinitis should be considered when interpreting or when defining FeNO reference values.

## Abbreviations

AR: allergic rhinitis; ATS/ERS: American Thoracic Society/European Respiratory Society; iNOS: inducible Nitric Oxide Synthase; NAR: Non-Allergic Rhinitis; SPT: Skin Prick Tests.

## Competing interests

DL and AMP declare no competing interests. JAF and TJ received an unrestricted grant from Aerocrine for the development of a FeNO interpretation aid tool (http://feno.med.up.pt).

## Authors' contributions

DL, TJ, JAF contributed to the design of the study, acquisition, interpretation and analysis of data and in the manuscript draft and review. AMP participated in the acquisition and interpretation of the data and in the manuscript draft and review. All authors read and approved the final manuscript.

## Supplementary Material

Additional file 1 **Tables with information on characteristics of the included studies and their samples**. Table S1. Studies' characteristics regarding country, smoking habits FeNO equipment used and study design; Table S2. Definitions of healthy groups and number of participants per group in included studies. Click here for file

Additional file 2 **meta-analysis comparing FeNO values in atopic and healthy children; in allergic rhinitis and healthy adults; and in allergic rhinitis and healthy children**. Figure S1. Meta-analysis of studies comparing FeNO values in Atopic and Healthy children; Figure S2. Meta-analysis of studies comparing FeNO values in Allergic Rhinitis and Healthy adults; Figure S3. Meta-analysis of studies comparing FeNO values in Allergic Rhinitis and Healthy children. Click here for file
