# Supplementary material for: Effects of atopy and rhinitis on exhaled nitric oxide values - a systematic review
Source: Clin Transl Allergy. 2011 Aug 17;1:8. doi: 10.1186/2045-7022-1-8 (PMC3339369; doi:10.1186/2045-7022-1-8)
Supplement: Additional file 1 — Tables with information on characteristics of the included studies and their samples. Table S1. Studies' characteristics regarding country, smoking habits FeNO equipment used and study design; Table S2. Definitions of healthy groups and number of participants per group in included studies. [file 2045-7022-1-8-S1.DOCX]

**Additional File 1**

Title: Tables S1-S2

Description: This file contains 2 tables with information on characteristics of the included studies and their samples.

**Table S1**. Studies’ characteristics regarding country, smoking habits FeNO equipment used and study design. NI: No information. Niox/Niox Mino is a product from Aerocrine AB, Sweden; Sievers is a product from GE Infrastucture, USA; Model LR2000 Logan Research is a product from Logan Research, Rochester, UK; Eco Medics Analyser CLD is a product from Eco Medics AG, Switzerland

| **Article** | **Title** | **Country** | **Smoking habits? (No Smokers)** | **FeNO Equipment** | **Study Design** |
| --- | --- | --- | --- | --- | --- |
| **Aronsson, 2005** | Allergic rhinitis with or without concomitant asthma: difference in perception of dyspnoea and levels of fractional exhaled nitric oxide | Sweden | No | Niox/Niox Mino | Prospective, Observational |
| **Berlyne, 2000** | A comparison of exhaled nitric oxide and induced sputum as markers of airway inflammation | Canada | No | Sievers | Cross Sectional |
| **Cardinale, 2005** | Exhaled nitric oxide, total serum IgE and allergic sensitization in childhood asthma and allergic rhinitis | Italy | No | Sievers | Cross Sectional |
| **Cibella, 2008** | Factors that influence exhaled nitric oxide in Italian schoolchildren | Italy | NI | Sievers | Cross Sectional |
| **Gratziou, 2008** | Breath markers of oxidative stress and airway inflammation in seasonal allergic rhinitis | Greece | No | Model LR2000, Logan Research | Prospective |
| **Hervás, 2008** | Differences in exhaled nitric oxide in atopic children | Spain | NI | Sievers | Cross Sectional |
| **Hung, 2007** | Montelukast decreased exhaled nitric oxide in children with perennial allergic rhinitis | China | NI | Niox/Niox Mino | Randomized Controlled |
| **Kosticas, 2008** | Portable exhaled nitric oxide as a screening tool for asthma in young adults during pollen season | Greece | Yes (76) | Niox/Niox Mino | Cross Sectional |
| **Malmberg, 2006** | Exhaled nitric oxide in healthy nonatopic school-age children: Determinants and height-adjusted reference values | Finland | Yes (10) | Niox/Niox Mino | Cross Sectional |
| **Manson, 2009** | Nasal CpG oligodeoxynucleotide administration induces a local inflammatory response in nonallergic individuals | Sweden | NI | Niox/Niox Mino | Cross Sectional |
| **Marcucci, 2007** | Lower airway inflammation before and after house dust mite nasal challenge: an age and allergen exposure-related phenomenon | Italy | NI | Sievers | Cross Sectional |
| **Olin, 2004** | Exhaled nitric oxide: Relation to sensitization and respiratory symptoms | Sweden | No | Eco Medics Analyser CLD | Cross Sectional |
| **Prieto, 2002 (Jun)** | Exhaled nitric oxide and bronchial responsiveness to adenosine 5-monophosphate in subjects with allergic rhinitis | Spain | No | Niox/Niox Mino | Cross Sectional |
| **Prieto, 2002 (Sep)** | Modifications of airway responsiveness to adenosine 5-monophosphate and exhaled nitric oxide concentrations after the pollen season in subjects with pollen-induced rhinitis | Spain | No | Niox/Niox Mino | Cross Sectional |
| **Profita, 2006** | Noninvasive methods for the detection of upper and lower airway inflammation in atopic children | Italy | NI | Niox/Niox Mino | Cross Sectional |
| **Rolla, 2007** | Diagnostic classification of persistent rhinitis and its relationship to exhaled nitric oxide and asthma: a clinical study of a consecutive series of patients | Italy | No | Niox/Niox Mino | Cross Sectional |
| **Rouhos, 2008** | Atopic sensitization to common allergens without symptoms or signs of airway disorders does not increase exhaled nitric oxide | Finland | No | Sievers | Cross Sectional |
| **Saito, 2004** | Exhaled nitric oxide as a marker of airway inflammation for an epidemiologic study in schoolchildren | Japan | NI | Sievers | Cross Sectional |
| **Tanou, 2009** | Inflammatory and oxidative stress biomarkers in allergic rhinitis: the effect of smoking | Greece | Yes (35) | Niox/Niox Mino | Cross Sectional |

**Table S2.** Definitions of healthy groups and number of participants per group in included studies. NI: No information; AR – Allergic Rhinitis; NAR – Non-Allergic Rhinitis

| **First Author** | **Healthy/Controls Definition** | **Number of Healthy/ Controls** | **Number of atopic** | **Number of AR** | **Number of NAR** |
| --- | --- | --- | --- | --- | --- |
| **Aronsson, 2005** | “Subjects who did not report a history of rhinitis or asthma, with negative SPTs, without hyper-responsiveness to metacholine” | 11 | - | 18 | - |
| **Berlyne, 2000** | “Subjects without symptoms, an FEV1/VC of 70% or greater, and a methacoline PC20 of greater than 16 mg/mL, without atopy” | 22 | 28 | - | - |
| **Cardinale, 2005** | (Subjects with) “No history of airway disease, allergy or significant medical illness and not taking any medication.” | 25 | - | 41 | - |
| **Cibella, 2008** | (Subjects) “Without asthma (positive answer to "Have you ever had asthma diagnosed by a physician") or rhinitis.” | 94 | 38 | 63 | 94 |
| **Gratziou, 2008** | “Healthy non-atopic volunteers” | 16 | - | 11 | - |
| **Hervás, 2008** | NI | 15 | 15 | 15 | - |
| **Hung, 2007** | “Subjects who revealed no history of allergies or respiratory disease within 4 weeks prior to beginning the study” | 12 | - | 45 | - |
| **Kosticas, 2008** | “Subjects without respiratory symptoms (based in all negative answers to a questionary)” | 70 | - | 57 | - |
| **Malmberg, 2006** | “Subjects nonsmokers and non-atopic; without a history of asthma or allergic rhinitis or any other chronic respiratory disorder, or a history of wheeze during the last 12 months; not taking any regular medication; free from symptoms and signs of acute upper respiratory tract infection during 2 weeks prior to the assessment; with a normal lung function (deﬁned as forced expired volume in 1 sec (FEV1) within 2 SD of predicted)” | 114 | 24 | - | - |
| **Manson, 2009** | “Symptom-free subjects, with no history of AR and a negative SPT to the standard panel of allergens” | 10 | - | 10 | - |
| **Marcucci, 2007** | “Healthy non-atopic” | 10 | - | 15 | - |
| **Olin, 2004** | NI | 137 | 33 | 10 | 17 |
| **Prieto, 2002 (1)** | “Individuals with no history of asthma, allergic rhinitis, atopic eczema, or other relevant disease, and no medication and negative skin-prick test result for six common airborne allergens” | 10 | - | - | 14 |
| **Prieto, 2002 (2)** | “Subjects had no history of asthma, allergic rhinitis, atopic eczema, or other relevant disease, and were receiving no medications. All subjects were nonatopic, as defined by a negative skin test result for six common airborne allergens.” | 10 | - | 38 | - |
| **Profita, 2006** | “Subjects with no acute respiratory symptoms in the last 4 weeks” | 16 | - | 18 | - |
| **Rolla, 2007** | NI | 40 | - | 42 | 23 |
| **Rouhos, 2008** | Exclusion process of: current smokers and ex-smokers; subjects with: bronchodilator response of FEV1 ≥ 12%, PD15FEV1 ≤ 0.4 mg histamine, FEV1/FVC < 88% of predicted, physician-diagnosed asthma or symptoms suggesting asthma, other respiratory symptoms, other significant diseases (epilepsia and hemiparesis, cardiovascular disease requiring several medications) orallergic rhinitis. | 41 | 32 | 10 | - |
| **Saito, 2004** | “Non-atopic subjects, non-wheezers” | 176 | 139 | - | - |
| **Tanou, 2009** | “Subjects presenting low levels of serum total non-speciﬁc IgE and presenting no reaction to the tested allergens” | 30 | - | 40 | - |
