# Supplementary material for: Effects of atopy and rhinitis on exhaled nitric oxide values - a systematic review
Source: Clin Transl Allergy. 2011 Aug 17;1:8. doi: 10.1186/2045-7022-1-8 (PMC3339369; doi:10.1186/2045-7022-1-8)
Supplement: Additional file 2 — meta-analysis comparing FeNO values in atopic and healthy children; in allergic rhinitis and healthy adults; and in allergic rhinitis and healthy children. Figure S1. Meta-analysis of studies comparing FeNO values in Atopic and Healthy children; Figure S2. Meta-analysis of studies comparing FeNO values in Allergic Rhinitis and Healthy adults; Figure S3. Meta-analysis of studies comparing FeNO values in Allergic Rhinitis and Healthy children. [file 2045-7022-1-8-S2.DOCX]

**Additional File 2**

Title: Meta-analysis

Description: This file contains 3 meta-analysis comparing FeNO values in atopic and healthy children; in allergic rhinitis and healthy adults; and in allergic rhinitis and healthy children.

**Figure S1.** Meta-analysis of studies comparing FeNO values in Atopic and Healthy children.

**Figure S2.** Meta-analysis of studies comparing FeNO values in Allergic Rhinitis and Healthy adults. DS: During Season; OS: Out of Season.

**Figure S3**. Meta-analysis of studies comparing FeNO values in Allergic Rhinitis and Healthy children.
